# Innovative immune mechanisms and antioxidative therapies of intervertebral disc degeneration

**DOI:** 10.3389/fbioe.2022.1023877

**Published:** 2022-10-10

**Authors:** Bingqian Wei, Yingjing Zhao, Weihang Li, Shilei Zhang, Ming Yan, Zebing Hu, Bo Gao

**Affiliations:** ^1^ Institute of Orthopedic Surgery, Xijing Hospital, Air Force Medical University, Xi’an, China; ^2^ Basic Medical College, Air Force Medical University, Xi’an, China; ^3^ Department of Critical Care Medicine, Nanjing First Hospital, Nanjing Medical University, Nanjing, China; ^4^ The Key Laboratory of Aerospace Medicine, Ministry of Education, Air Force Medical University, Xi’an, China

**Keywords:** intervertebral disc degeneration(IDD), inflammatory response, antioxidative therapies, macrophages, cytokines

## Abstract

Intervertebral disc degeneration (IDD) is the basic pathological process of many degenerative diseases of the spine, characterized by series of symptoms, among which low back pain (LBP) is the most common symptom that patients suffer a lot, which not only makes patients and individual families bear a huge pain and psychological burden, but also consumes a lot of medical resources. IDD is usually thought to be relevant with various factors such as genetic predisposition, trauma and aging, and IDD progression is tightly relevant with structural and functional alterations. IDD processes are caused by series of pathological processes, including oxidative stress, matrix decomposition, inflammatory reaction, apoptosis, abnormal proliferation, cell senescence, autophagy as well as sepsis process, among which the oxidative stress and inflammatory response are considered as key link in IDD. The production and clearance of ROS are tightly connected with oxidative stress, which would further simulate various signaling pathways. The phenotype of disc cells could change from matrix anabolism-to matrix catabolism- and proinflammatory-phenotype during IDD. Recent decades, with the relevant reports about oxidative stress and inflammatory response in IDD increasing gradually, the mechanisms researches have attracted much more attention. Consequently, this study focused on the indispensable roles of the oxidative stress and inflammatory response (especially macrophages and cytokines) to illustrate the origin, development, and deterioration of IDD, aiming to provide novel insights in the molecular mechanisms as well as significant clinical values for IDD.

## 1 Introduction

### 1.1 Background

Intervertebral disc degeneration (IDD) is the basic pathological process of many degenerative diseases of the spine, which is clinically manifested as spinal stenosis, vertebral segment instability, lumbar and leg pain, cervical spondylosis, intervertebral disc herniation etc., and the patient’s nerve roots and spinal cord are compressed to produce a series of complications, among which low back pain (LBP) is the most common symptom that patients suffer a lot, which not only makes patients and individual families bear a huge pain and psychological burden, but also consumes a lot of medical resources (Li et al., 2021a). Existed studies believed that biomechanical property is the main factor to keep the spinal flexibility as well as mechanical stability (Lam et al., 2011), among them pilots are the main victims that usually suffer from serious damage to the cervical and lumbar vertebrae (Albermann et al., 2020). But increasing researches have suggested that in addition to biomechanical factors, the autoimmune system also behaves an essential role in the degeneration process of IDD, including oxidative stress, exosomes, inflammatory cytokines, etc. (Peng, 2008; Johnson et al., 2015; Li et al., 2021a; Xiang et al., 2022). Therefore, this study combined and focused on the researches about the roles of oxidative stress, cytokines mediums and inflammatory reaction on IDD in recent decades, aiming to provide novel insights in the molecular mechanisms, as well as guidance and references for the potential therapeutic strategies for IDD.

### 1.2 Structures of intervertebral disc

Intervertebral disc (IVD) is primarily comprised of three parts, including nucleus pulposus (NP), annulus fibrous ring (AF) and cartilage endplate (CEP). NP is a highly watery, jelly-shaped tissue in the middle part, with a dense network of collagen fibers located inside the NP, each layer of collagen fibers covered with a mucopolysacly protein complex and chondroitin sulfate, so that the nucleus of the medullary can bind to water, acting as a fulcrum in adjacent vertebral activity, like a ball, moving forward and backward with the flexion and extension of the spine (Le Maitre et al., 2007). AF is surrounded by almost concentric rings of fibers, containing three layers: outer, medium and inner, the outer layer is composed of collagen fiber bands, and the inner layer is composed of fiber cartilage bands, which are tightly attached to the CEP to keep the stability of the spine. The CEP contained fibro-chondrite, located between the body of the vertebrae above and lower, which could withstand pressure and prevent the vertebrae from being overloaded by pressure (Kadow et al., 2015). Under physiological state, due to the special location of the NP that is surrounded by CEP and fibrous rings, the NP located in a closed space and is isolated from the immune systematic reactions, which is served as the largest immune privilege organ within body (Sun et al., 2020a).

### 1.3 Oxidative stress

Redox homeostasis is essential for the maintenance of physiological process in many cellular activities, the dysregulation of redox homeostasis would influence human health and is directly related to pathological conditions (Akanji et al., 2021). Within this process, oxidative stress is regarded as the imbalance situation between two different states, including the generation of active metabolites and free radicals [also known as reactive oxygen species (ROS) or oxidants] and the removal of the above substances by antioxidants. This imbalance has serious damage not only to biomolecules and cells, but also to the entire organism (Ďuračková, 2010). Increasing evidences have illustrated the pivotal roles of oxidative stress in the pathogenesis of various diseases including degenerative skeletal diseases (Kimball et al., 2021; Kulkarni et al., 2021; Zhao et al., 2021).

ROS is the product of normal cellular metabolism, which is mostly produced by mitochondrial respiratory chains (Poyton et al., 2009), which can respond to changes of environmental conditions inside/outside the cell and react accordingly to signaling pathway modulation. In endogenous metabolic reactions, ROS is mostly the normal product of molecular oxygen biological reduction, such as superoxide anions (O_2_−), hydroxyl radicals (OH•), hydrogen peroxide (H_2_O_2_), and organic peroxides (Fridovich, 1978). However, the excessive accumulation of ROS by continuous environmental stress or other pathological processes *in vivo*, could induce and promote oxidative stress, which may cause damage to biological macromolecules like nucleic acids, carbohydrates proteins and lipids, and finally destroyed the cell structure and functions of body (Kumar et al., 2008; Fang et al., 2009; Venza et al., 2015).

Existed studies have reported the complex antioxidative system with different functions participated to protect the body from excess oxidants damage, among which the antioxidants behaved essential roles, such as glutathione peroxidase (GPx), superoxide dismutase (SOD), catalase and glutathione reductase (enzymatic antioxidants), and also vitamin C/D and glutathione (GSH) (non-enzymatic antioxidants) (Sies, 1991). The theoretical foundation have also provided guidelines in the treatment IDD (Feng et al., 2017).

### 1.4 Autoimmune theory

In 1977, Gertzbein proposed the hypothesis of autoimmune theory in IDD through a large number of animal experiments and clinical studies, he found that degenerative disk overexpressed Toll-like receptors Toll-2 and Toll-4, which could be stimulated by the products of extracellular matrix and enhance the inflammatory and immune response; and the evidence for autoimmune mechanism in IDD came from the existence of cellular reaction through both lymphocyte transformation test and leukocyte-migration inhibition test (Gertzbein, 1977; Gertzbein et al., 1977). Bobechko and Hirshl reported the responses of regional lymph nodes of rabbits with ears implanted by autologous disc material (Bobechko and Hirsch, 1965). In the physiological state, the NP tissue is isolated from the body’s immune monitor due to the encapsulation of the AF ring and the CEP, and has no direct contact with the peripheral circulation. When the IVD is damaged or injured, the NP tissue breaks through the encirclement of the AF ring and the posterior longitudinal ligament. While during repair process, the neovascular vessel grows into the NP tissue so that the NP tissue is in close contact with the immune system (Binch et al., 2014; Li et al., 2022). Glycoproteins and β proteins are served as antigens in the stroma of the NP, and the body may produce immune response under the continuous stimulation of these antigens, which is also involved the IVD of other segments, further causing degeneration of the IVD (Gertzbein, 1977).

## 2 Oxidative stress and antioxidative therapies in IDD

### 2.1 Interactions between oxidative stress/inflammation and IDD

As previously mentioned, there remained imbalance states between ROS production and clearance in degenerative discs. There were a great deal of evidences that ROS was widely participated in metabolic modulation, signaling transduction, cell death, cell aging, phenotypic transformation of IVD cells, which jointly to regulate the activity and functions of disc and further accelerate the development of IDD (Feng et al., 2017). Oxidative stress reactions caused by excessive ROS could further stimulate a variety of aberrant signaling pathways in IVD cells, like MAPK and NF-κB pathways, and ultimately strengthening both local and systemic oxidative stress (Li et al., 2021b; Chen et al., 2022; Zhu et al., 2022). The phenotype of IVD cells changed from matrix anabolism phenotype to matrix catabolism and pro-inflammatory phenotype, suggesting significant matrix loss and increased inflammation in IVD circumstances. In addition, IVD cells also secreted chemokines that enhanced inflammation by recruiting immune cells into the disc, which further secrete more cytokines and chemokines in turn, thus worsening the activity and functions of IVD cells, finally leading a vicious circle (Risbud and Shapiro, 2014; Li et al., 2022).

### 2.2 Antioxidative therapies in IDD

Glutathione (GSH) is a natural peptide and it’s the primary antioxidant in living cells. Yang et al. (2014a) suggested that GSH could effectively prevent the harmful effects of H2O2 or IL-1β in NP, thereby inhibiting ROS production, apoptosis as well as matrix decomposition in human NP cells. N-Acetylcysteine (NAC) served as the progenitor of GSH, have been studied to prevent the progression of IDD by lowering ROS levels and weakening MAPK signaling pathway mediated by ROS or TNF-α in AF cells (Suzuki et al., 2015). At the same time, premature aging of IVD cells has also been improved: treatment with NAC could reverse the NP cell apoptosis and ECM degradation; within needle-induced disc degeneration in rat model, oral NAC inhibited oxidative stress, stromal catabolism, and inflammation. Additionally, commonly served as a supplement, NAC had few toxic side effects reports, which could be used as a great option for IVD degeneration treatment (Seol et al., 2021; Bai et al., 2022).

Resveratrol (RSV) was a polyphenol product which mainly existed in vegetation. Research by Li et al. reported the effective anabolic effects of RSV about IVD homeostasis on bovine: RSV could inhibit MMP-13 expression and promote proteoglycan synthesis in NP cells, which could also reverse the catabolism roles of IL-1 and bFGF which were responsible for oxidative stress, proliferation and apoptosis. They also elucidated the responses of multiple downstream cascade molecules after RSV activation, which made the roles of RSV better understand, such as anti-inflammatory, antioxidant and antiproliferative effects, etc. (Li et al., 2008). Among them, RSV could protect NP cells from degradation by elevating cell survival and functions, which may be related to the suppression of JAK/STAT3 phosphorylation and the decrease of IL-6 products (Wu et al., 2021). Previous studies have reported that epigallocatechin 3-gallate (EGCG), a polyphenol product existed in thea viridis, could inhibit the inflammation response of diagnostic cells *in vivo* or *in vitro*, which also displayed analgesic activity for disc-relevant radiculopathy in animals (Krupkova et al., 2014). Studies by Krupkova et al. have shown that EGCG activated PI3K/AKT signaling pathway, which was an important pro-activation mechanism under deadly oxidative stress (Krupkova et al., 2016). Combined with these biological effects and functions of EGCG mentioned above, we believed that EGCG could be further used to develop new therapies for oxidative stress in degenerative disc disease.

Pyrroloquinoline (PQQ) was a redox cofactor of bacterial dehydrogenase, which had the potential to eliminate ROS production and reduce apoptotic process. The results of Yang et al. showed that PQQ enabled NP cells to exhibit high cell viability, which inhibited excessive generation of ROS in rat NP cells induced by H_2_O_2_, and thereby protecting NP cells from apoptosis. It also antagonized downregulation of type II collagen and agrican in H_2_O_2_-induced NP cells. Therefore, PQQ could be regarded as potential lead compound in the prevention of IDD (Yang et al., 2015).

## 3 The roles of macrophages in the development of IDD

### 3.1 Origin and differentiation of macrophages

Mouse LY6C^hi^/LY6C^low^ cells are the same as human CD14^+^/CD14^low^CD16^+^ monocyte/macrophage sub populations, respectively (Sun et al., 2020b). Present hematopoietic project pointed out that hematopoietic stem cell (HSC)–derived GMPs promoted the generation of MDPs and MDPs further enhanced cMoPs production, which was the direct progenitor of LY6C^hi^ and LY6C^low^ macrophages, and LY6C^hi^ macrophages circulating in blood mostly relied on the CCR2-dependent extravasation (Ginhoux and Jung, 2014; Guilliams et al., 2018).

**FIGURE 1:**
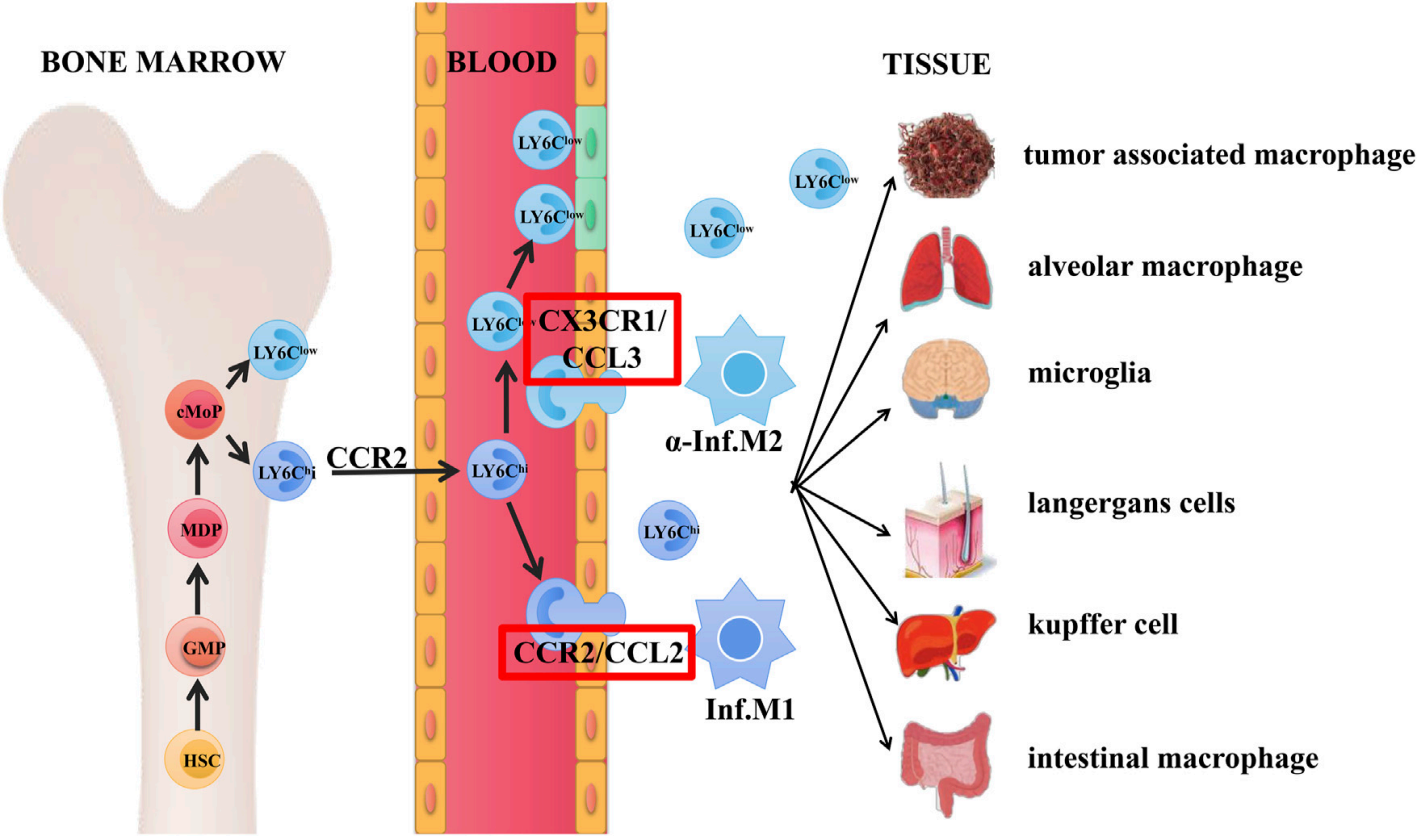
Origin and differentiation of macrophages. Hematopoietic stem cell (HSC)–derived GMPs promoted the generation of MDPs and subsequently enhanced cMoPs production, which was the direct progenitors of LY6C^hi^ and LY6C^low^ monocytes. LY6C^hi^ monocytes circulating in the blood relied on the CCR2-dependent extravasation.

Within the homeostatic state, LY6C^hi^ and LY6C^low^ macrophage sub populations in the circulation generated developmental continuum with different functions: Macrophage-like LY6C^low^ cells in the blood monitored the endothelial surface and enrolled neutrophils as needed to manipulate the reconstruction process; they were recruited together and then differentiated into different states like M2Mϕ, thereby secreting anti-inflammatory cytokines to promote tissue repair. By contrast, LY6C^hi^ monocytes served as “canonical monocytes”, were clustered into inflammatory region and could be regarded as the progenitors of peripheral mononuclear phagocytes to behave roles. They would differentiate into mature inflammatory M1Mϕ, contributing to tissue degradation and T cell activation (Shi and Pamer, 2011; Yang et al., 2014b; Ginhoux and Jung, 2014; Varol et al., 2015). LY6C^hi^ macrophage could transform into diversity of cells under certain circumstances, like langerhans cells, microglia cells, kupffer cells, alveolar macrophages, and intestinal macrophage in different tissues. Besides, macrophages could also transform into tumor-associated macrophages with tumor development and metastasis functions (Gentek et al., 2014; Varol et al., 2015), the detailed developmental process of these subtypes and possible signaling pathways were shown in Figure 1.

As mentioned above, in the steady state, macrophage-like LY6C^low^ cells acted as patrol and monitored the intravascular dynamics, while LY6C^hi^ macrophages acted as “canonical monocytes” and were clustered into inflammation region. Ly6C^low^ macrophages were recruited together by interactive roles of CX3CR1/CCL3 pair through LAF/ICAM1 signaling pathway and thus transformed into M2Mϕ state, secreting anti-inflammatory cytokines and finally enhanced the tissue restore (Cros et al., 2010). In the aspect of vascular inflammation, LY6C^hi^ monocytes were activated and infiltrated into tissue by interaction roles of CCR2/CCL2(MPC-1) through VLA-1/VCAM1 transduction, which were then transformed into inflammatory M1Mϕ state, finally contributing to tissue deterioration and T cell stimulation (Yang et al., 2014b).

### 3.2 Different roles of M1 and M2 in IDD

Basically, phenotype with high levels of IL-12, IL-23, and low levels of IL-10 were mainly existed in M1 cells, which were effector molecules like nitrogen intermediates, ROS, and inflammatory mediators. As inducers and effector cells, M1 macrophages participated in the polarization Th1 response, and regulated the tolerance of parasites and neoplasms. On the contrary, M2 macrophages mainly possessed low levels of IL-12, IL-23, and high levels of IL-10 phenotypes, and their generated inflammatory cytokines chiefly depended on different signaling pathways. Generally, M2 cells were involved in the polarization Th2 response, parasite clearance, inflammation inhibition, tissue remodeling promotion, angiogenesis, tumor progression, and immunomodulation.

At present, the researches of macrophages in degenerative disc were mainly focused on M1, M2a, and M2c, the cell markers were defined as CCR7^+^, CD206^+^, and CD163^+^, respectively. Studies have shown that CCR7^+^, CD163^+^, and CD206^+^ cells were existed in human IVD. CCR7^+^, and CD163^+^ cells increased with the degree of IVD deterioration; M1 and M2c macrophage phenotypes were highly expressed in the IVD region with irregular and defective structures (Nakazawa et al., 2018). CCR7^+^ M1 phenotype was known to secrete pro-inflammatory cytokines like TNF-α and IL-1β; and CD163^+^ M2c phenotype produced high levels of MMP required for ECM remodeling; while CD206^+^ M2a phenotype with anti-inflammatory functions was often connected with the last stages of wound healing, tissue restore, ECM decomposition as well as fibrosis (Ni et al., 2019; Li et al., 2021c). Thus, the accumulation in M1-and M2c-like cells (not M2a) kept pro-inflammatory and remodeling state in IDD without transitioning to wound healing. In addition, all three macrophage markers in epes increased significantly with the degree of deterioration, while only CCR7^+^ in NP increased significantly, and macrophage markers did not exist in the AF region (Nakazawa et al., 2018). Therefore, the trend between the positive rate percentage and degradation grade of macrophage markers did not always match the changes in each region across IVD. Compared to normal CEP cells as well as the irregular morphology and organization of these cells, the degradation trends of CCR7^+^, CD163^+^, and CD206^+^ macrophages in CEP gave evidence for the hypothesis that exogenous macrophages infiltrated through the CEP, especially through cell migration from the CEP to NP region, which has already been well described (Jimbo et al., 2005; Jia et al., 2020).

CHI3L1, namely chitinase 3-like 1 protein, was a secretory glycoprotein which promoted tumor infiltration and migration in various neoplasms by elevating the expression levels of matrix metalloproteinase (MMPs) family genes (Studer et al., 2011; Fang and Jiang, 2016; Jin et al., 2019). Research also reported that the expression of CHI3L1was highly increased in M2a compared to other types of macrophages (Wang et al., 2019). The roles between M2a cells and CHI3L1 in IDD was demonstrated by Li et al. through rat IDD models: M2a cells generated CHI3L1 protein and acted on the underlying receptor IL-13R, mediating ECM degradation in NP cells through ERK and JNK-specific pathways rather than p38 pathways. In this process, the recombinant CHI3L1 significantly enhanced the expression of MMP3 and MMP9, and inhibited agglomerated sugar and collagen II expression in NP cells. Besides, the roles of CHI3L1 in degeneration of IDD was behaved upon concentration- and time-dependent manner (Li et al., 2021d).

### 3.3 Potential treatment of IDD targeting macrophages

Wang et al. (2020a) have shown that macrophages could activate T cells through the JAK-STAT signaling pathway, causing a cascade of inflammatory responses. More than 50 cytokines, including IFN-γ, IL-2, IL-6, IL-12, and IL-23, which were dependent on the JAK-STAT pathway, and drugs that inhibited the JAK protein could simultaneously prevent the activity of these cytokines. Therefore, drugs destroying communication networks induced by cytokines may be an efficient way to treat these diseases including IDD.

There remained four FDA-approved JAK inhibitors currently: Ruxolitinib, Tofacitinib, Baracitinib and Upadacitinib. Other JAK inhibitors were in different preclinical and clinical stages, which were mainly used to treat multiple autoimmune and autoinflammatory diseases (Panés et al., 2017; Ma et al., 2019). A preclinical *in-vivo* testing of Tofacitinib in bovine IDD model by Li et al. have reported the potential roles of anti-inflammatory drug Tofacitinib in ameliorating IDD, by downregulating IL-1β, IL-6, IL-8, MMP1, MMP3 in NP tissue, and MMP3, COX2 (cyclooxygenase-2), NGF (nerve growth factor) in AF tissue, thereby neutralizing pro-inflammatory and catabolic circumstance in IDD model (Li et al., 2020). Besides, served as a potent pan-JAK inhibitor, Tofacitinib could inhibit M1 macrophages polarization by suppressing the activation of STAT1, which have shown prospects in the treatment of corneal allograft rejection (Yu et al., 2022). A case report by Yi et al. suggested that Baracitinib was an option in the maintenance therapy of macrophage activation syndrome, which was potentially beneficial to prevent the recurrence (Yi et al., 2022). However, the relevant researches of these JAK inhibitors on IDD remained insufficient. Considering the close connections between these JAK inhibitors and macrophages, as well as the macrophage roles in IDD, the relationships between JAK inhibitors and IDD were subtle. Based on the promising prospects of ameliorating NP cells from degradation through JAK inhibition by recent study, we believed JAK inhibitors would also be a kind of potential compounds in the treatment of IDD targeting macrophages (Wu et al., 2021). More researches about the detailed mechanisms of these JAK inhibitors in the treatment of IDD still need further exploration.

## 4 The relationships between cytokines and IDD

### 4.1 IL-17

In recent years, accumulating evidences suggested that IL-17, also known as IL-17A, behaved pivotal roles in the development of IDD, which was produced by T helper cell 17 (Th17), a sub population of CD4 T cells (Liu et al., 2019). Unlike the classic Th1 and Th2 lineages, IL-17 was absent in human normal AF cells and lowly expressed in normal NP cells, while especially increased in human degenerative NP cells. More importantly, the expression levels of IL-17 in IVD increased with the IDD degree, displaying that IL-17 may be an effective indicator reflecting severity of IDD (Tan et al., 2022).

**FIGURE 2:**
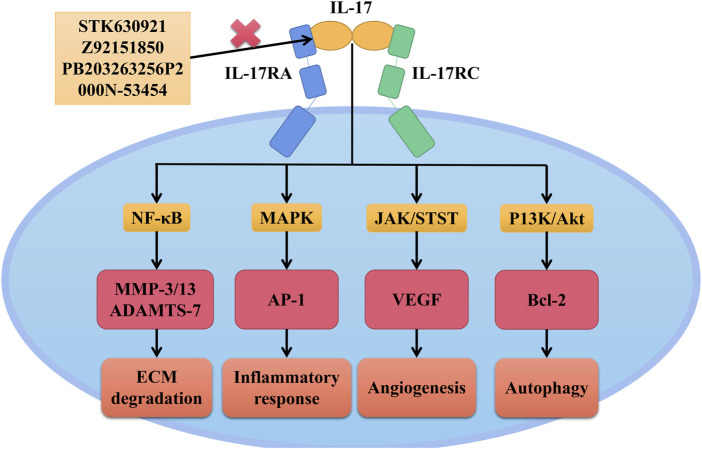
Interactive roles of IL-17 in IDD. After IL-17 binding to its receptors, IL-17 could increase the levels of MMP-3, MMP-13 and ADAMTS-7 by motivating the nuclear translocation of NF-κB, thus promoting ECM degradation; IL-17 stimulated the MAPK/AP-1 transduction to modulate the generation of pro-inflammatory substances. IL-17 could induce angiogenesis by upregulating the JAK/STAT/VEGF signal axis, and prevented NP cell from autophagy by promoting the PI3K/AKT/Bcl-2 signaling cascade. STK630921, Z92151850, PB203263256, and P2000N-53454 were small molecular drugs blocking the interaction roles between IL-17 and IL-17RA.

Studies have suggested that IL-17 could increase the levels of MMP-3, MMP-13, and ADAMTS-7 by motivating the nuclear translocation of NF-κB, thus promoting ECM degradation (Tan et al., 2022); IL-17 also stimulated the MAPK/AP-1 transduction to modulate the generation of pro-inflammatory substances (Li et al., 2016); Besides, IL-17 could induce angiogenesis by upregulating the JAK/STAT/VEGF signal axis, and also prevented NP cells from autophagy by promoting the PI3K/AKT/Bcl-2 signaling cascade (Hu et al., 2017). These findings implied the essential roles of IL-17 in the development of IDD. STK630921, Z92151850, PB203263256, and P2000N-53454 were small molecular drugs blocking the interaction roles between IL-17 and IL-17RA (Suyama et al., 2018; Tan et al., 2022). The application of these inhibitors has been shown to inhibit ECM decomposition and pro-inflammatory substances production in IL-17-induced rat NP cells under hypoxic conditions, among which STK630921 displayed the most effective results (Tan et al., 2022). Consequently, IL-17 was a potential biomarker reflecting the severity of IDD degree, and inhibitors which blocked IL-17 and IL-17RA could be served as an important direction for future clinical drug discovery. The detailed mechanism roles of the current IL-17 inhibitors were illustrated in Figure 2.

### 4.2 IL-6

**FIGURE 3:**
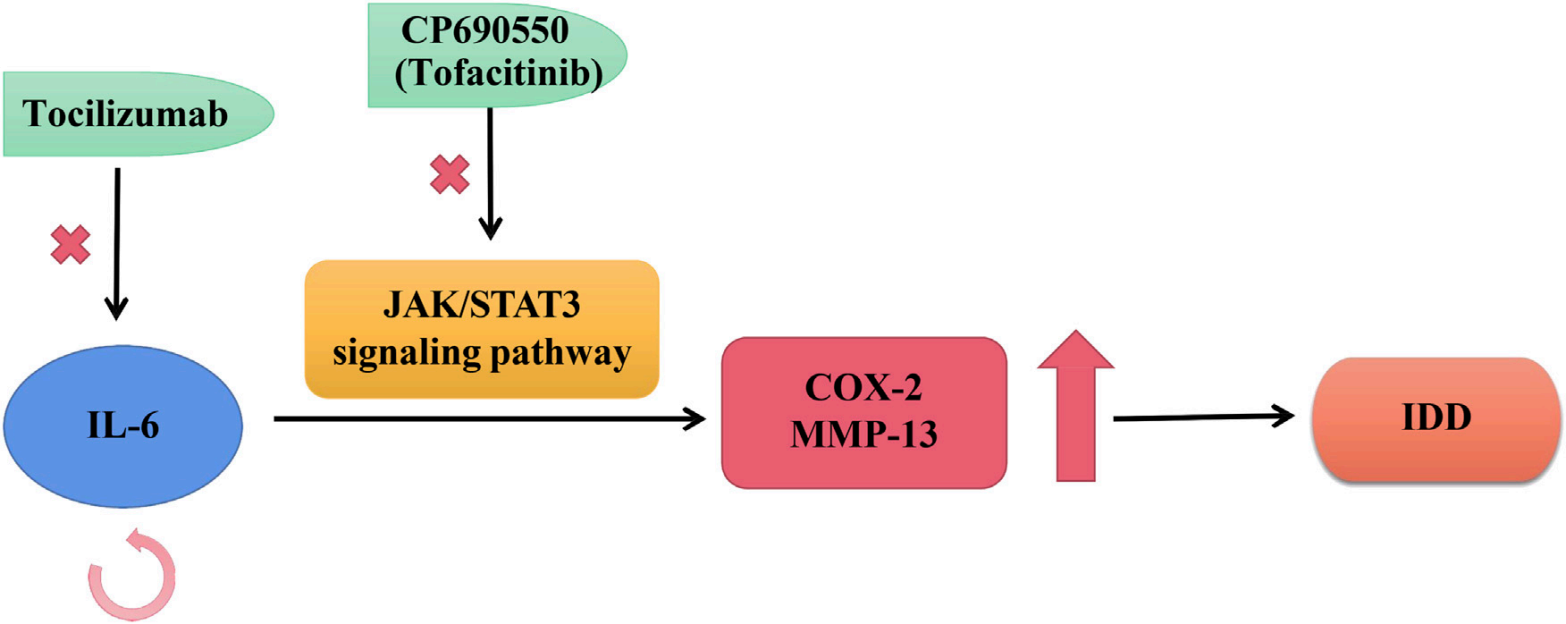
Roles of IL-6 in IVD. IL-6 self-stimulated expression and enhances the expression of mediators like COX-2 and MMP-13, thus exacerbating IDD progression through the JAK/STAT3 transduction. Tocilizumab was a kind of humanized monoclonal antibody against human IL-6 receptors; CP690550 was an oral JAK antagonist, also called tofacitinib, was being developed to treat auto-immune diseases like RA.

Suzuki et al. reported a high expression situation of IL-6 during degenerative IVD in rats and humans, which self-amplified its own expression and promoted the expression levels of mediators like COX-2 and MMP-13, thereby exacerbating IVD degeneration through the JAK/STAT3 signaling pathway (Suzuki et al., 2017). CP690550, also called Tofacitinib, was an oral JAK antagonist, which was currently being developed as the effective approach for RA (rheumatoid arthritis) and other auto-immune diseases (Ohtori et al., 2012). CP690550 also significantly inhibited IL-6-mediated gene expression to ameliorate IDD by pharmacological inhibition of JAK3 activity (Ohtori et al., 2012; Suzuki et al., 2017). These findings strongly demonstrated a causal relationship between the IL-6/JAK/STAT3 cascade reactions and the development of IDD. Therefore, this intracellular pathway could provide evidence for targeted treatment of preventing IDD. There is evidence that epidural Tocilizumab was effective in relieving low back pain for patients with IDD (Ohtori et al., 2012), as shown in Figure 3.

Moreover, recent clinical data suggested that IL-6 could also be a reasonable and efficient target in bone degenerative disease in addition to IDD. The humanized monoclonal antibody Tocilizumab against human IL-6 receptors has been widely applied in the treatment of RA, indicating the essential roles of target therapy based on IL-6 in skeletal destructions (Smolen et al., 2008).

### 4.3 IL-β

Numerous studies have found that IL-1β was involved in the development of IDD through different ways. IL-1β behaved roles in creating pro-inflammatory/degenerative IVD conditions; When exposure to IL-1β, the production of mononuclear cytochemical aspiration proteins-1, IL-6, IL-8, and prostaglandin E2 increased significantly (Yuan et al., 2018). Additionally, exposure to IL-1β stimulated the production of IL-17 in degenerative IVD cells, which has been elaborated above (Gruber et al., 2013). IL-1β could also improve its own generation by promoting the activation of NLRP3 inflammasomes, while the NLRP3 inflammation and NF-κB activation could be blocked by IKK-β selective inhibitor Bay11-7082 (Zhang et al., 2017; Chen et al., 2020). Consequently, BAY11-7082 may also be a potential aspect of IDD clinical drug therapy research, the detailed mechanisms need further exploration.

In terms of oxidative stress, Liu et al. demonstrated that IL-1β could significantly increase ROS and catabolic activity within mouse vertebral bone marrow stromal cells (vBMSCs), this process could be effectively prevented by fullerol (Liu et al., 2013). Therefore, considering the connections about IL-1β and fullerol in IDD and ROS respectively, fullerol may be served as a valid biological therapy to treat IDD, further experimental studies need to focus on fullerol to validate these effects.

### 4.4 TNF-α

Existed researches have demonstrated that TNF-α could promote the accumulation of IL-6, IL-8 and IL-17 in AF cells, stimulating the secretion of inflammatory substances such as NO and PGE2, and aggravated the inflammatory response, the same as IL-1β (Huang et al., 2020; Qi et al., 2020). Furthermore, Liu et al. have found that the TNF-α stimulated IVD cells could expression enhance the expression levels of CCL3, CCL20, CXCL2, and CXCL5 genes, which were associated with the ECM decomposition, damage, inflammatory reactions, and the regulation of apoptosis (Gabr et al., 2011). Besides, TNF-α could improve the levels of intercellular adhesion molecules (ICAM-1) in human IVD cells, which was one of the most important pairs of adhesion molecules (Wang et al., 2020b). Thus, more relationships between TNF-α, ICAM-1 and IDD were worth studying.

## 5 Summary and outlook

The pathogenesis of IDD is complex, involving multiple cellular activities and multiple regulatory pathways. In this review, we discussed the pivotal roles of oxidative stress and inflammatory response in IDD and potential therapies with oral drugs. There remained imbalance between ROS production and clearance in degenerative discs, oxidative stress reactions caused by excessive ROS could further stimulate a variety of signaling pathways and ultimately strengthen both local and systemic oxidative stress. Besides, natural antioxidants such as GSH, RSV and PQQ all provided promising prospects in the treatment of IDD, which were used to develop novel therapies in IDD.

Additionally, macrophages and cytokines also played important roles in inflammatory response through various signaling pathways, providing a great number of targets to treat IDD including JAK inhibitors, IKK-β selective inhibitor and humanized monoclonal antibody against human IL-6 andIL-17 receptors, etc. However, the drug treatment of IDD based on the targets mentioned above remained little studied, thus there is still a long way to go to comprehensively explore the potential values of these drugs in the treatment of IDD.
